# Angiosarcoma of liver: A case report of a 56-year old with a highly aggressive tumor

**DOI:** 10.1016/j.ijscr.2025.111502

**Published:** 2025-06-16

**Authors:** M. Siddique Khan, Abdal Qureshi, Mahad Younas, Saba Noor, Manal Gohar, Aimal Khan

**Affiliations:** a Department of Surgery, Khyber Teaching Hospital, Peshawar, Pakistan; b Khyber Medical College, Peshawar, Pakistan

**Keywords:** Hepatic angiosarcoma, Hepatic vascular lesions, Left lateral segmentectomy, Case report

## Abstract

**Introduction and importance:**

Hepatic angiosarcoma is a rare and aggressive tumor of the vascular endothelial cells with a poor prognosis. Due to its nonspecific symptoms and rapid progression, early diagnosis poses a significant challenge. Histopathology and immunohistochemistry are essential for confirmation; however, treatment options remain limited.

**Case presentation:**

A 56-year-old male presented to us with right upper quadrant pain, anemia, and mild ascites. Computed Tomography (CT) imaging revealed multiple vascular liver and spleen lesions, initially suspected as metastatic. Tumor markers and Fine Needle Aspiration Cytology (FNAC) of a thyroid nodule were performed which turned out to be inconclusive. Due to internal hemorrhage, an emergency left lateral segmentectomy was performed. Cluster of Differentiation 34 (CD34) and Erythroblast transformation specific regulated gene (ERG) histopathology confirmed hepatic angiosarcoma. The patient was stable postoperatively and referred for oncology care. However, the patient deteriorated soon after and died due to massive intra-abdominal hemorrhage before initiating chemotherapy.

**Clinical discussion:**

Hepatic angiosarcoma is often multifocal with rapid metastasis, making complete resection almost impossible. CT and Magnetic Resonance Imaging (MRI) aid in detection but histopathology with vascular markers CD34 and ERG are essential for definitive diagnosis. In light of lack of treatment options surgical resection offers the best survival for localized disease, while chemotherapy and Transarterial Chemoembolization (TACE) remain good palliative options. With a median survival of 6 months, the prognosis of this disease is highly dismal.

**Conclusion:**

Hepatic angiosarcoma is a highly fatal condition with limited treatment options. Histopathology is crucial for diagnosis, and surgery remains the best option for localized cases. Early recognition is key to improving outcomes.

## Highlights

• Hepatic angiosarcoma is associated with poor prognosis and limited treatment.
• CD34 and ERG histopathology is essential for definitive diagnosis.
• Surgical resection remains the primary treatment option.
• Early diagnosis and intervention are crucial for improved patient outcomes.

## 1 Introduction

Angiosarcoma is an uncommon and aggressive cancer that originates in the endothelial cells lining the blood or lymphatic vessels. It most commonly appears in the head and neck area, with the breast being the next most frequent site, and has an extremely disappointing prognosis. The liver is identified as the fifth most common location for this malignancy [1]. Due to its aggressive behavior and varied presentation, early detection and customized treatment approaches are crucial for enhancing patient outcomes. Historically, primary hepatic angiosarcoma has been linked to exposure to toxins such as thorotrast, vinyl chloride, anabolic steroids, and exogenous steroids. Despite these associations, the majority of cases lack a definitive causative factor. The nonspecific symptoms of this disease include weight loss, abdominal distention, jaundice, abdominal pain, and fatigue. Additionally, patients may be asymptomatic, with the disease often being incidentally discovered during imaging studies conducted for other reasons, and has a characteristically poor prognosis [2].

Our case highlights the diagnostic challenges and clinical presentation of liver angiosarcoma. Given its rarity and nonspecific symptoms, early recognition is crucial. Documenting this case contributes to understanding the disease and underscores the importance of prompt evaluation and management for improved patient outcomes. This case report has been reported in line with the SCARE checklist [3].

## 2 Case description

**Fig. 1:**
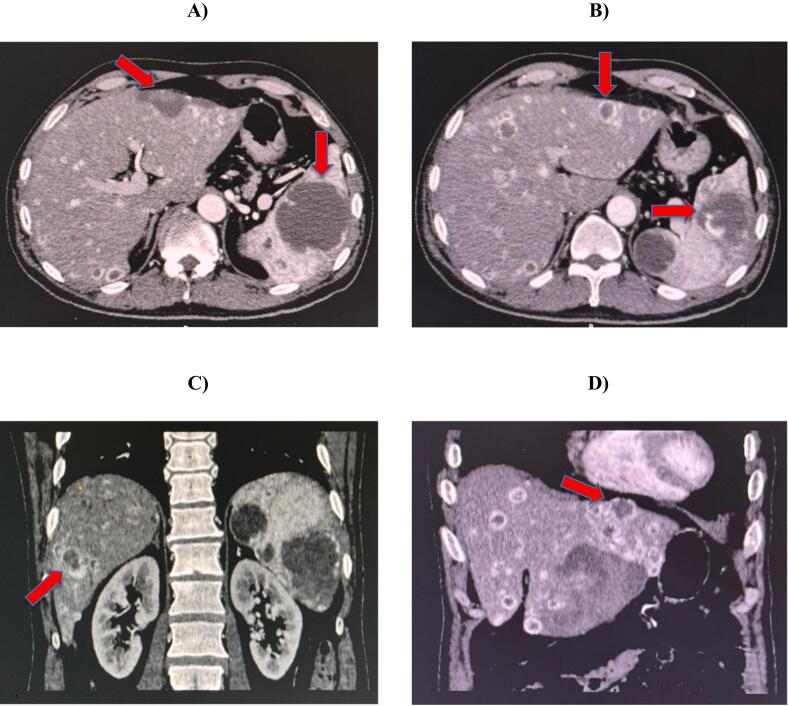
A (venous phase) & B (arterial phase): the axial sections of the CT scan show multiple contrast-enhanced heterogeneous lesions scattered throughout the liver more pronounced in the left lateral segment having peripheral arterial enhancement with central hypodensities. Similar multiple lesions are found in the spleen having peripheral arterial enhancement more pronounced in the arterial phase. (C, D): the coronal view showing similar findings with multiple, large heterogenous lesions having central hypodensities more evident in the left lateral segment of the liver (venous phase).

A 56-year-old male patient presented to us with right upper quadrant pain, low hemoglobin levels, mild abdominopelvic ascites, and multiple, diffused solid and high vascular lesions of variable sizes in the spleen and liver on a Computed Tomography (CT) scan (Fig. 1). The highly vascular, multifocal hepatic and splenic metastatic deposits raised a possible suspicion of a distant metastasis with the primary focal point either being in the thyroid gland or the kidneys apart from other areas. All tumor markers, including Alpha-Fetoprotein (AFP), Cancer Antigen 19–9 (CA19–9), and Carcinoembryonic Antigen (CEA) were normal. Ultrasound neck revealed a hypodense nodule in the thyroid gland. Fine Needle Aspiration Cytology (FNAC) was performed but was inconclusive and Thyroglobulin test was normal. Urine routine examination showed numerous red cells but malignant cytology was negative. The patient was referred to an interventional radiologist for angio-embolization, but was not possible due to the presence of multiple lesions.

**Fig. 2:**
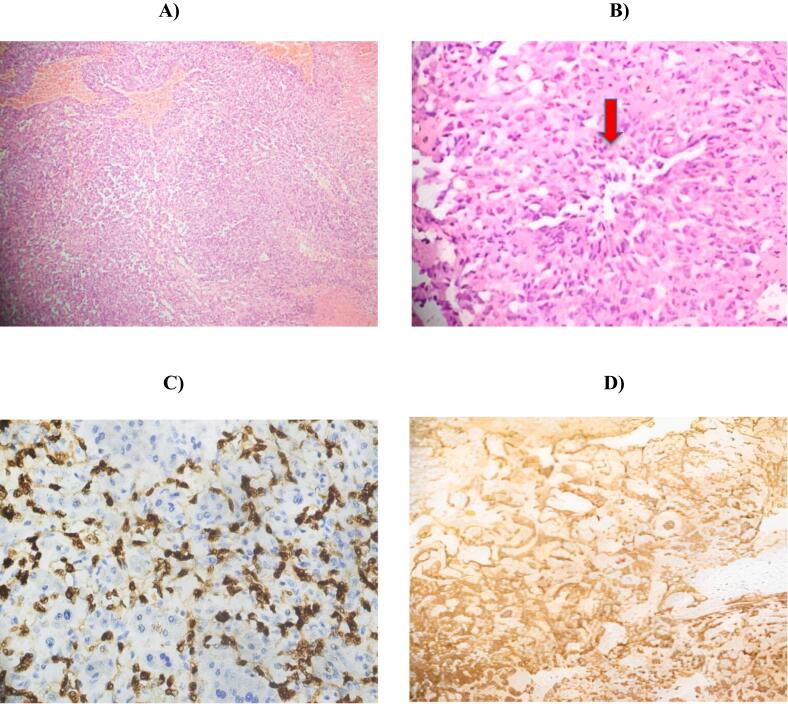
(A, B): Hematoxylin and Eosin (H&E) stain showing irregularly shaped anastomosing vascular channels lined by atypical, multilayered endothelial cells with nuclear pleomorphism (see arrow) (C): Immunohistochemistry positive staining for ERG (D): Immunohistochemistry positive staining for CD34.

Meanwhile, the patient developed internal abdominal bleeding due to hemorrhage from one of the lesions. An emergency left lateral segmentectomy (segments II & III) was performed and the resected specimen was sent for histopathological assessment. Biopsy reports intimated primary angiosarcoma of the liver. Immunohistochemistry tests for Cluster of Differentiation 34 (CD34) and Erythroblast transformation specific regulated gene (ERG) markers depicted positive stains (Fig. 2).

Our patient was a school teacher belonging to the northern region of Swat, Pakistan. He enjoyed a healthy lifestyle with an unremarkable past medical and surgical history. He also had no prior exposure to any industrial toxins, which was deciphered from the detailed patient history, that could have potentially predisposed him to developing hepatic angiosarcoma. Post-operatively the patient was substantially stable and was managed with supportive medications. Furthermore, he was referred to the oncology department for adjuvant therapy.

On a follow up of two weeks, the patient was supposed to begin his chemotherapy but he suddenly became unstable and eventually died due to profuse bleeding in his local hospital. This work is in concordance with the guidelines of the Surgical Case Report (SCARE) criteria [3].

## 3 Discussion

Liver angiosarcoma is a rare malignancy, accounting for approximately 0.1–2 % of all primary liver tumors. This aggressive cancer predominantly affects men in their sixth or seventh decade of life, with a male-to-female incidence ratio of 3:1. The prognosis is generally poor, with a high mortality rate and median survival of 6 months [4]. Accurately diagnosing this tumor can be challenging, particularly in the absence of a history of exposure to specific carcinogens. The symptoms, clinical signs, and results of liver function tests at the time of presentation frequently lack specificity, further complicating the diagnostic process. Due to similar cytological features, Angiosarcomas must be distinguished from epithelioid sarcomas and malignant melanomas. Epithelioid sarcomas (CD34- and ERG-positive) lack Cluster of Differentiation (CD31) and Integrase Interactor 1/SWI/SNF-related matrix-associated actin-dependent regulator of chromatin subfamily B member 1 (INI/SMARCB1), unlike angiosarcomas, while melanomas are S100, SRY-related HMG-box 10 (SOX10), and melan-A positive but negative for vascular markers necessitating immunohistochemical panels for accurate diagnosis [5].

In resource-limited settings like Pakistan, challenges with timely diagnosis are amplified due to scarcity of advanced imaging modalities such as Positron Emission Tomography (PET) scans. Limited availability of specialized interventional radiology and pathology expertise further contribute to the diagnostic dilemmas of the patient and clinicians, significantly compromising the timely management of rare malignancies like hepatic angiosarcoma. Additionally, exposure to toxins such polyvinyl chloride has been linked to TP53 mutations whereas KRAS-2 mutations have been identified in cancer cells from thorotrast exposure with both type of molecular alterations having been associated with liver angiosarcoma [1]. However, neither the patient was found to be exposed to such toxins nor molecular tests could be performed due to our limited resources setup.

The onset of angiosarcoma is mainly but not always specified to late adulthood and more often in men than women. The presenting symptoms markedly vary from patient to patient making early diagnosis extremely onerous. However, literature explains some common symptoms including abdominal discomfort, general malaise, weight loss, fatigue, anorexia, and severe recurrent pain in the upper right quadrant of the abdomen. Patients may also present with mild abdomino-pelvic ascites, jaundice, distension, and low hemoglobin (Hb) levels in case of ongoing internal hemorrhages of the lesions. Differential diagnoses for such cases include hemangioendothelioma, hepatocellular carcinoma, intrahepatic cholangiocarcinoma, hepatic adenoma, and hepatic angiosarcoma. Radiological diagnostic procedures such as CT scans can be helpful but are mainly inconclusive and non-diagnostic in such cases. The definitive diagnoses can only be established upon the histopathological and immunohistochemical findings in a liver biopsy [6].

Primary hepatic angiosarcoma is highly aggressive with rapid progressing and early metastasis. Complete hepatic resection is effective for single lesions but not feasible in metastatic cases. Liver transplants are discouraged due to high recurrence and rapid progression. While no established chemotherapy exists, 5-FU-carboplatin with doxorubicin or ifosfamide has shown some efficacy, and Transarterial chemoembolization (TACE) can be used for palliation or bleeding control [1,2]. Primary hepatic angiosarcoma (PHA) is often multicentric and diffused, but surgery is recommended for singular or oligometastatic lesions. Cytotoxic chemotherapy is the primary systemic treatment for multifocal or metastatic PHA [7].

With respect to our case, while the patient was being investigated for the primary cause of metastasis, he suddenly started to bleed and became unstable and a left lateral segmentectomy had to be performed in an emergency situation. Post-operation, a histopathological assessment of the specimen was performed which returned positive for tumor markers CD34 and ERG. To definitively rule out other possible primary metastases, a thyroglobulin test and FNAC were performed which returned normal and hence thyroid carcinoma was ruled out. For renal cell carcinoma, a urine cytology for malignant cell as well as a CT scan were performed which were both negative. Additionally, the patient was scheduled for a PET scan but could not be performed since the patient became unstable prior to it and the aforementioned surgery was done.

The best course of angiosarcoma treatment inclines towards a combined approach of surgical resection followed by chemotherapy. In 2000, Timaran et al. [4] reported the 10-year survival of an angiosarcoma patient after a complete surgical resection. Similarly, Arima-Iwasa et al. [8] showed a patient living healthily 16 months on after undergoing hepatic resection for angiosarcoma without any significant signs of recurrence.

In one of the largest case series to date comprising 44 cases of hepatic angiosarcoma in a multicentric, international setup Wilson et al. [9] show evidentially the poor prognosis of hepatic angiosarcoma patients with a median survival period of 5.8 months. Surgical resection was demonstrated to be the predominantly successful treatment option for any chances of long-term survival with patients undergoing the procedure having an overall survival of 33 months, with two of the patients having a greater than 5-year survival. On the other hand, Park et al. [10] argued transcatheter arterial chemoembolization (TACE) not only be the primary procedure for restricting bleeding after hemorrhage of the hepatic angiosarcoma lesions but also be potentially effective in treating the disease itself. However, no well-fortified evidence was provided in this regard by either the said study or any piece of literature.

What makes our case unique is the fatal course of events that the disease took. Firstly, our patient had a non-existent exposure to any of the causative industrial toxins mentioned in literature. Secondly and most importantly, the acute deterioration of our patient's condition within two weeks of surgery highlights a highly aggressive behavior not commonly documented in literature. For instance, Wilson et al. reported improved patient survival outcomes for those who underwent surgical resection whereas our patient did not meet both the time frame regarding the median survival period for the cohort as well as survival data of those who underwent surgical resection. This stark difference underscores not only the heterogenic behavior of liver angiosarcoma but also calls for a deeper look into factors such as tumor microenvironment that may influence prognosis in such a disease. Our case, however, did conform to certain aspects of the said study and other literature such as the vague and inconclusive symptoms with which the patient presented and the extensive liver involvement and marked metastasis at the time of diagnosis.

## 4 Conclusions

Hepatic angiosarcoma is a rare malignant, aggressive condition that is more common in males. CT scan helps in making a diagnosis but histopathology with CD31 and CD34 staining is the gold standard. While curative options are limited, prompt surgical resection in localized disease may offer temporary disease control or improvement. This case highlights the importance of early diagnosis, high degree clinical suspicion, and timely intervention even in the absence of traditional risk factors.

## Informed consent

An informed consent was taken from the patient after thorough explanation of the purpose of the research.

## Ethical approval

An ethical certificate of approval was obtained from the Institutional Research and Ethical Review Board (IREB) for the conductance of this work.

## Funding

No financial support was provided by any funding agency in public, commercial or not-for-profit sector.

## Author contribution

**Dr. M Siddique Khan:** Conceptualization, Investigation, Project administration, Supervision, Visualization, Writing – review & editing. **Abdal Qureshi:** Data curation, Investigation, Methodology, Validation, Writing – original draft, Writing – review & editing. **Mahad Younas:** Data curation, Investigation, Methodology, Validation, Writing – original draft, Writing – review & editing. **Saba Noor:** Data curation, Investigation, Methodology, Validation, Writing – original draft, Writing – review & editing. **Dr. Manal Gohar:** Data curation, Investigation, Methodology, Validation, Writing – original draft, Writing – review & editing. **Aimal Khan:** Data curation, Methodology, Writing – original draft.

## Guarantor

Abdal Qureshi.

## Research registration number

N/A.

## Conflict of interest statement

The authors declare no conflict of interest.
